# The impact of postoperative agitated delirium on dementia in surgical patients

**DOI:** 10.1093/braincomms/fcae076

**Published:** 2024-03-19

**Authors:** Mingyang Sun, Wan-Ming Chen, Szu-Yuan Wu, Jiaqiang Zhang

**Affiliations:** Department of Anesthesiology and Perioperative Medicine, Henan Provincial People’s Hospital, People’s Hospital of Zhengzhou University, Zhengzhou 450003, China; Graduate Institute of Business Administration, College of Management, Fu Jen Catholic University, Taipei 24205, Taiwan; Artificial Intelligence Development Center, Fu Jen Catholic University, Taipei 24205, Taiwan; Graduate Institute of Business Administration, College of Management, Fu Jen Catholic University, Taipei 24205, Taiwan; Artificial Intelligence Development Center, Fu Jen Catholic University, Taipei 24205, Taiwan; Department of Food Nutrition and Health Biotechnology, College of Medical and Health Science, Asia University, Taichung 41354, Taiwan; Big Data Center, Lo-Hsu Medical Foundation, Lotung Poh-Ai Hospital, Yilan 265501, Taiwan; Division of Radiation Oncology, Lo-Hsu Medical Foundation, Lotung Poh-Ai Hospital, Yilan 265501, Taiwan; Department of Healthcare Administration, College of Medical and Health Science, Asia University, Taichung 41354, Taiwan; Cancer Center, Lo-Hsu Medical Foundation, Lotung Poh-Ai Hospital, Yilan 41354, Taiwan; Centers for Regional Anesthesia and Pain Medicine, Taipei Municipal Wan Fang Hospital, Taipei Medical University, Taipei 110301, Taiwan; Department of Management, College of Management, Fo Guang University, Yilan 265501, Taiwan; Department of Anesthesiology and Perioperative Medicine, Henan Provincial People’s Hospital, People’s Hospital of Zhengzhou University, Zhengzhou 450003, China

**Keywords:** postoperative agitated delirium, dementia, incidence rate, PSM

## Abstract

This study investigates the association between postoperative agitated delirium and the risk of dementia in patients who were cognitively intact before undergoing major inpatient surgery. The study included inpatients aged 20 years or older who underwent major surgery requiring general, epidural, or spinal anaesthesia and hospitalization for over one day in Taiwan between 2008 and 2018. Patients were categorized into two groups based on the presence or absence of postoperative agitated delirium. Propensity score matching was conducted to balance various covariates known to influence dementia risk. The final analysis included 10 932 patients (5466 in each group). Multivariate Cox regression analysis was performed to assess the risk of dementia, and incidence rates and incidence rate ratios were calculated. After Propensity score matching, the study cohort comprised 5467 patients without postoperative agitated delirium and 5467 patients with postoperative agitated delirium. In the multivariate Cox regression analysis, the adjusted hazard ratio for dementia were 1.26 (95% confidence intervals, 1.08–1.46; *P* = 0.003) in the postoperative agitated delirium group compared to the no postoperative agitated delirium group. The incidence rates of dementia was significantly higher in patients with postoperative agitated delirium (97.65 versus 70.85 per 10 000 person-years), with an incidence rate ratio of 1.21 (95% CI: 1.04–1.40). Our study demonstrates a substantial rise in dementia incidence linked to postoperative agitated delirium. These findings stress the need for effective prevention and management strategies. Addressing this issue emerges as a vital clinical approach to reduce subsequent dementia risk, with broad implications for enhancing overall perioperative patient outcomes.

## Teaser

In a study of 10 932 post-surgery patients, Sun *et al*. found a 1.26-fold higher risk of dementia in those with postoperative agitated delirium (adjusted hazard ratio 1.26, 95% confidence interval 1.08–1.46, *P* = 0.003), emphasizing the need for better prevention and management.

## Graphical Abstract

**Graphical Abstract:**
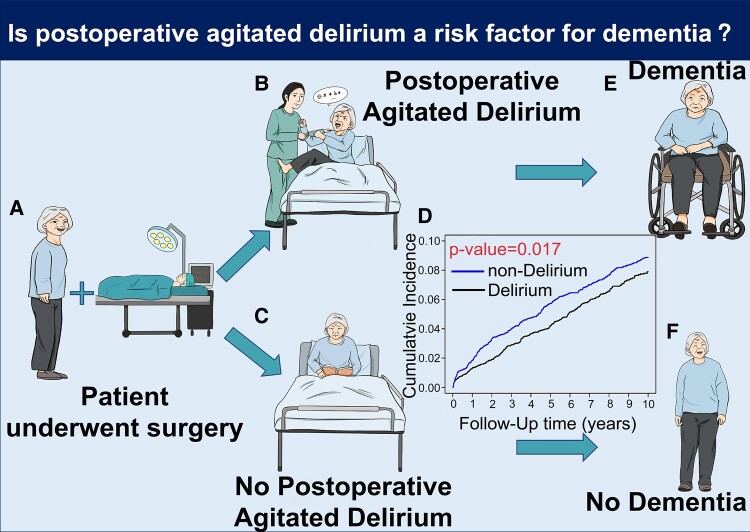

## Introduction

Delirium stands as a prevalent and distressing complication encountered in the perioperative phase. Postoperative delirium (POD) typically manifests within 24–72 h following surgery and is characterized by a state of acute confusion that is generally reversible.^1^ This condition is characterized by fluctuating attention and awareness, disorientation, memory impairment, perceptual disturbances and disorganized thinking.^2^ Of note, postoperative agitated delirium is a particularly prominent presentation in clinical practice. Dementia, a progressive and insidious neurodegenerative disorder, is defined by chronic cognitive decline in one or more domains, ultimately impinging upon an individual's ability to maintain independence in daily life. This stands in stark contrast to the more acute and temporary nature of postoperative agitated delirium.^3^ It stands as a leading cause of cognitive impairment, primarily affecting older populations, imposing a significant burden on both patients’ quality of life and societal resources.^4^ Yet, the intricate interplay between POD and dementia remains an area of considerable ambiguity.^5-9^ While previous investigations have indicated that dementia serves as a primary risk factor for delirium, conversely, delirium emerges as an independent risk factor for subsequent dementia development.^10^ These complex associations underscore the pressing need for comprehensive research endeavours to elucidate the underlying mechanisms and clinical implications of this intricate relationship.

Understanding the link between POD and dementia is crucial due to established evidence that delirium significantly increases the risk of subsequent dementia, particularly in the elderly.^11^ Various mechanisms, such as neurotoxicity, inflammatory cascades, chronic stress responses and neuronal damage, contribute to this association.^10,12-14^ While investigations have explored POD occurring concurrently with pre-existing dementia,^15^ the precise role of POD in the genesis of dementia in younger patients without preoperative cognitive dysfunction remains unclear. Clarifying this relationship has important implications for clinical practice and enhances our comprehension of cognitive health trajectories.

In this investigation, our primary aim is to elucidate the intricate relationship between the onset of dementia in patients who initially exhibited no cognitive impairments before undergoing surgery but subsequently developed agitated delirium necessitating medical intervention. To achieve this goal, we embarked on a meticulously designed head-to-head propensity score–matched study. The overarching objective of this study is 2-fold: first, to harmonize and balance the diverse covariates associated with our study population; and second, to rigorously assess the risk of dementia in surgical patients, drawing a clear distinction between those who experienced postoperative agitated delirium requiring pharmacological intervention and those who did not. This inquiry endeavours to provide crucial insights into the potential contribution of postoperative agitated delirium to the development of dementia in individuals without pre-existing cognitive deficits, thereby enhancing our understanding of the interplay between these clinical entities and informing future clinical practice and research directions.

## Materials and methods

### Study population

This investigation harnessed the extensive dataset from the Taiwan National Health Insurance Research Database (NHIRD) spanning from January 2008 through December 2018, and the ensuing follow-up period extended until 31 December 2020. Comprising an impressive cohort of around 27.38 million beneficiaries, the NHIRD encompasses a comprehensive repository of registration records and unaltered claims data, rendering it a formidable resource. Within these encrypted NHIRD archives, exhaustive details pertaining to both outpatient and inpatient claims, including patient identification numbers, birthdates, gender and diagnoses coded according to the International Classification of Diseases, Ninth and Tenth Revision, Clinical Modification (ICD-9-CM and ICD-10-CM), as well as treatment specifics, financial outlays, dates of hospitalization commencement and cessation and even mortality dates were meticulously extracted.^16^ These datasets were interconnected using patient identification numbers, providing us with a comprehensive view of the healthcare landscape.

### Inclusion and exclusion criteria

In our investigation, we included individuals aged 20 years or older who underwent significant inpatient surgery necessitating general, epidural, or spinal anaesthesia and required hospitalization exceeding one day during the period spanning from 2008 to 2018 in Taiwan.^17^ Patients undergoing cardiac or vascular surgeries were judiciously excluded due to their heightened susceptibility to POD and the concomitant elevated risk of mortality, a pivotal competing factor in our primary outcome of dementia. Additionally, patients with pre-existing diagnoses of dementia, cognitive impairment, Parkinson's disease, or prior occurrences of POD before the index date, which was defined as the major surgery date, were diligently excluded. Any patients with incomplete baseline information, as delineated in Supplemental Table 1, were similarly excluded from our study.

The enrolled patient cohort was stratified into two distinct groups contingent upon their postoperative status regarding agitated delirium following major surgery: Group 1 comprised 245 619 patients who remained free of postoperative agitated delirium, while Group 2 encompassed 5467 patients diagnosed with postoperative agitated delirium (refer to Supplemental Table 1 for details). The primary objective of our study was to elucidate the influence of postoperative agitated delirium on the subsequent risk of dementia in these distinct groups. To address the potential impact of mortality on our primary endpoint, we adeptly employed competing risk analysis methodologies.^18-20^

### Propensity score matching and covariates

To ensure the reliability of our study in assessing dementia risk associated with postoperative agitated delirium in major surgery patients, we meticulously applied statistical techniques to minimize the impact of confounding factors. Specifically, we conducted meticulous 1:1 propensity score matching (PSM) with a narrow caliper of 0.1 for multiple variables known to impact dementia in major surgery patients, as delineated in Supplemental Table 1.^21,22^ We initially identified a cohort of 251 086 patients undergoing major surgery from 2008 to 2018. After rigorous PSM, our final cohort included 10 932 patients (5466 in each group). These variables encompassed a wide spectrum, including age, gender, income levels, urbanization status, surgical procedure types, elective surgery status, American Society of Anesthesiologists (ASA) physical status, anaesthesia type (general or regional), anaesthesia duration, concurrent comorbidities (comprising but not confined to diabetes, hypertension, hyperlipidaemia, coronary artery disease, stroke, depression, anxiety, heart failure, peripheral vascular disease, chronic obstructive pulmonary disease, atrial fibrillation, traumatic head injury, sleep disorders, alcohol-related liver diseases, frailty, vision or hearing impairments, anaemia), medication utilization (such as diphenhydramine, anticholinergics, benzodiazepines, gabapentinoids, opioids and other psychotropic drugs) and Charlson Comorbidity Index (CCI) scores. Subsequently, we harnessed Cox proportional hazards models to gauge the influence of postoperative agitated delirium on dementia risk in major surgery patients. Robust sandwich estimators were employed to account for clustering within matched sets.^22^ Multivariate Cox regression analyses were conducted to compute hazard ratios (HRs) associated with dementia risk following postoperative agitated delirium in major surgery patients. Comorbidities were identified via ICD-9-CM or ICD-10-CM codes as primary diagnoses in inpatient records or outpatient encounters occurring at least twice within a 1-year timeframe. For continuous variables, we reported means ± standard deviations when applicable. By meticulously employing these stringent statistical methodologies, our study furnishes a trustworthy appraisal of the dementia risk associated with postoperative agitated delirium in real-world settings among patients undergoing major surgery.

### Statistical analysis

To uphold the credibility of our results, we conducted all statistical analyses using SAS version 9.4 (SAS Institute, Cary, NC, USA) and meticulously controlled for potential confounding factors. We deemed statistical significance to be achieved at a threshold of *P* < 0.05, employing a two-tailed Wald test. We used the Kaplan–Meier method to estimate dementia risk and compared groups using the log-rank test. This test was stratified by matched sets within the PSM cohort, ensuring a thorough and reliable assessment of our findings.^23^

## Results

### Study cohorts before and after PSM

We identified a cohort comprising 251 086 patients who underwent major surgery during the period spanning 2008–2018. Among them, 97.8% (*n* = 245 619) did not manifest postoperative agitated delirium, while 2.2% (*n* = 5467) did (refer to Supplemental Table 1). Notably, the postoperative agitated delirium group exhibited a higher prevalence of elderly individuals, males, individuals with lower income levels, those who underwent neurosurgery or respiratory surgery, individuals subjected to emergency surgery, those with elevated ASA scores, lengthier anaesthesia durations, greater coexisting comorbidities, heightened medication usage and elevated CCI scores compared to their counterparts in the non-postoperative agitated delirium group.

Following meticulous PSM, our final analytical cohort comprised 10 932 patients (5466 in each group) who were subjected to further scrutiny. The PSM process effectively balanced key covariates. These included age, gender, income levels, urbanization, surgical types, elective surgical status, ASA physical status, anaesthesia types, anaesthesia duration, coexisting comorbidities, medication usage and CCI scores. Absolute standardized mean differences consistently remained below 0.1.^21,22^ Post PSM, the observed rates of dementia stood at 6.7% in the postoperative agitated delirium group and 5.1% in the no POD group (see Supplemental Table 1). It’s noteworthy that statistical significance was established at *P* < 0.05 utilizing a two-tailed Wald test.

### Cox proportional hazard models of dementia

Before applying PSM, and subsequent to meticulous adjustment for potential confounding factors encompassing age, gender, income levels, urbanization status, surgical procedure types, elective surgical status, ASA physical status, anaesthesia types, anaesthesia duration, coexisting comorbidities, medication usage and CCI scores, we assessed dementia HRs via Cox proportional hazards models. The outcomes unequivocally underscored that individuals who experienced postoperative agitated delirium faced a notably elevated risk of dementia in comparison to those who did not (adjusted HR, 1.27; 95% CI, 1.14–1.41; *P* < 0.001).

Subsequently, following the PSM procedure, we delved deeper into the link between postoperative agitated delirium and dementia risk employing a multivariate Cox regression analysis, as meticulously presented in Table 1. Both univariate and multivariate analyses consistently showed that postoperative agitated delirium significantly increased dementia risk in major surgery patients. Precisely, the adjusted hazard ratios (aHRs) (95% CIs) for dementia stood at 1.26 (95% CI, 1.08–1.46; *P* = 0.003) within the postoperative agitated delirium group compared to the non-postoperative agitated delirium group. These findings, encapsulated, stress that individuals encountering postoperative agitated delirium face a markedly amplified risk of dementia, even after meticulous adjustments for potential confounding variables. This underscores a robust association between postoperative agitated delirium and long-term cognitive outcomes in major surgery patients, underscoring the paramount significance of preventive and management strategies for this prevalent complication.

**Table 1:** A Cox proportional regression model of dementia risk in patients receiving non-cardiac major surgery with and without postoperative agitated delirium

	Before propensity scores patching					
	Crude HR(95%CI)		*P*-value	Adjusted HR^a^ (95%CI)		*P*-value
**No postoperative agitated delirium (ref.)**	1.00			1.00		
**Postoperative agitated delirium**	1.60	(1.45, 1.78)	<0.001	1.27	(1.14, 1.41)	<0.001

	After propensity scores patching					
	Crude HR(95%CI)		*P*-value	Adjusted HR^a^ (95%CI)		*P*-value
**No postoperative agitated delirium (ref.)**	1.00					
**Postoperative agitated delirium**	1.20	(1.03, 1.39)	0.017	1.26	(1.08, 1.46)	0.003

	Competing risk of death for propensity scores matching cohort					
	Crude HR(95%CI)		*P*-value	Adjusted HR^a^ (95%CI)		*P*-value
**No postoperative agitated delirium (ref.)**	1.00			1.00		
**Postoperative agitated delirium**	1.20	(1.03, 1.39)	0.017	1.24	(1.07, 1.45)	0.004

HR, hazard ratio; CI, confidence intervals; ref., reference group.

^a^Adjustment of age, sex, income levels, urbanization, surgical types, elective surgical status, ASA physical status, types of anaesthesia, duration of anaesthesia, coexisting comorbidities, medications use and CCI scores.

Our investigation centred on dementia as the primary endpoint, yet we acknowledge the presence of death as a competing event that can influence the likelihood of dementia development. In response, we conducted a comprehensive competing risk analysis, which adeptly accommodates the influence of death on dementia incidence.^18-20^ Through Cox regression HRs, we meticulously evaluated the nexus between dementia and assorted predictor variables while effectively accounting for the competing risk stemming from death. This approach yielded a more nuanced comprehension of the relationship between postoperative agitated delirium and dementia probability, factoring in the influence of death as a competing risk. Within our analysis of propensity score–matched patients, a substantial and statistically significant association emerged between postoperative agitated delirium and dementia risk. Adjusted HRs of 1.24 (95% CI, 1.07–1.45; *P* = 0.004) underscored the heightened dementia risk within the postoperative agitated delirium cohort relative to their counterparts without this complication.

### Postoperative agitated delirium and dementia risk in Major surgery patients

Table 2 elucidates the association between postoperative agitated delirium and the onset of dementia, as well as the corresponding incidence rate ratio, in patients undergoing non-cardiac major surgery. Before PSM, patients who developed postoperative agitated delirium exhibited a notably higher incidence rate (IR) of dementia in comparison to those who did not (97.61 versus 60.38 per 10 000 person-years). This disparity translated into an incidence rate ratio (IRR) of 1.62 (95% CI: 1.46–1.79).

**Table 2:** Incidence rate and ratio of dementia in patients receiving non-cardiac major surgery with and without postoperative agitated delirium

	Events	Person-years	Incidence rate 10 000 person year	Incidence rate ratio	95% CI for incidence rate ratio	*P-*value
**Before propensity scores patching**						
No postoperative agitated delirium (ref.)	12,251	2,029,052.0	60.38	1.00		
Postoperative agitated delirium	365	37,392.6	97.61	1.62	(1.46, 1.79)	<0.001
**After propensity scores patching**						
No postoperative agitated delirium (ref.)	279	48,570.0	70.85	1.00		
Postoperative agitated delirium	365	37,378.5	97.65	1.21	(1.04, 1.40)	0.013

CI, confidence intervals; ref., reference group.

Subsequent to PSM, the trend persisted, with patients experiencing postoperative agitated delirium showcasing a significantly heightened IR of dementia relative to their counterparts without this complication (97.65 versus 70.85 per 10 000 person-years). The corresponding IRR remained statistically significant at 1.21 (95% CI: 1.04–1.40). These compelling findings underscore that the incidence of dementia remains significantly elevated among individuals who encounter postoperative agitated delirium, even after meticulous adjustments for potential confounding factors via PSM.

### Kaplan–Maier curves of dementia

**Figure 1:**
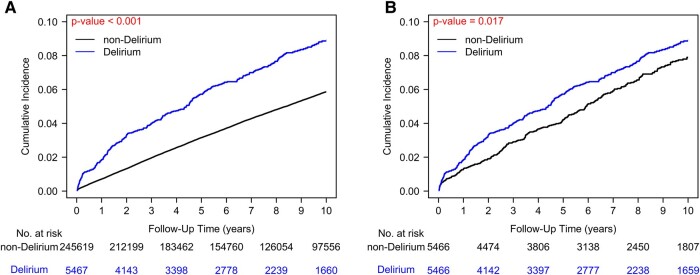
**Kaplan–Meier cumulative incidence curves of dementia in patients undergoing non-cardiac major surgery with and without postoperative agitated delirium.** Statistical significance was assessed using a stratified log-rank test (*P* < 0.001). (**A**) Before propensity scores match. (**B**) After propensity scores match.

We observed a notably elevated incidence rate of dementia within the postoperative agitated delirium group compared to the no POD group before PSM (Fig. 1A, *P* < 0.001). Among patients undergoing non-cardiac surgery, the 5-year cumulative dementia rates stood at 5.9% and 2.8% for the postoperative agitated delirium and no postoperative agitated delirium groups, respectively, before PSM.

Specifically, after PSM, we used Kaplan–Meier curves to visualize the occurrence of dementia in cohorts with and without postoperative agitated delirium among major surgery patients (Fig. 1B). The analysis demonstrated a significant difference in the overall incidence of dementia over time between individuals with and without postoperative agitated delirium (Fig. 1B, *P* = 0.017). In the post-PSM context, focusing on patients undergoing non-cardiac surgery, the 5-year cumulative dementia rates were 5.9 and 4.1% for the postoperative agitated delirium and no postoperative agitated delirium groups, respectively. In Fig. 1, the ‘number at risk’ below the plots indicates the count of participants actively engaged in the study, who have not experienced the specified event (dementia) or been censored (exited the study, lost to follow-up) at each respective time point.

## Discussion

Postoperative delirium, a complex neuropsychiatric syndrome characterized by transient cognitive impairment and confusion following surgical procedures, has emerged as a pivotal contributor to the development of dementia, particularly among elderly patients.^15,24,25^ A comprehensive exploration of this phenomenon has unveiled that advanced age, pre-existing cognitive deficits and the nature of the surgical intervention play pivotal roles in exacerbating this risk.^15,24,25^ These seminal findings underscore the critical importance of healthcare practitioners’ vigilance in recognizing the prodromal symptoms of POD, as elucidated in the research conducted at esteemed institutions.^15,24,25^ It is imperative that healthcare providers deploy targeted strategies to mitigate the incidence of POD, thereby potentially ameliorating the long-term risk of dementia in vulnerable patient populations. However, it is essential to note that POD manifests in distinct types.^26^ Among these, agitated delirium is readily observable. Consequently, past research has faced challenges in comprehensively assessing the relationship between POD and the subsequent risk of dementia,^8,9^ particularly concerning which delirium type holds the greatest association with future dementia development. As of now, the association between the risk of dementia and POD remains a subject of ongoing debate.^8,9^ While some previous studies have suggested that patients with cognitive impairment or dementia may be at an elevated risk of developing POD,^27^ a critical question remains unanswered: can postoperative agitated delirium, in turn, promote the onset of postoperative dementia? This study, including 10 932 patients after PSM, offers valuable insights. It found that the incidence of dementia in those who developed agitated delirium post-surgery was 6.7%, compared to 5.1% in patients without agitated POD. Statistical significance, as determined by a two-tailed Wald test, supports these findings. Importantly, this research meticulously adjusted for various factors, including age, comorbidities and surgical characteristics, revealing that postoperative agitated delirium represents a notable risk factor for postoperative dementia. The aHRs indicated a 26% increased risk of dementia (95% CI, 1.08–1.46; *P* = 0.003) in patients who experienced postoperative agitated delirium compared to those who did not. This underscores the need for continued research to elucidate the intricate relationship between delirium types and their impact on the development of dementia.

In our exploration of the association between distinct types of POD and the subsequent risk of postoperative dementia, previous studies have not provided clear distinctions among different POD types.^8,9,28,29^ Therefore, the specific POD type contributing to a heightened risk of dementia remains unclear. Our study stands as a pioneering effort, being the first to establish a definitive link between postoperative agitated delirium and the risk, incidence rate and incidence rate ratio of postoperative dementia. Unlike previous research,^8,9,28,29^ our investigation sheds light on the substantial impact of postoperative agitated delirium on the risk of developing postoperative dementia. This novel insight is essential for understanding the nuanced relationship between different POD types and their distinct influences on dementia outcomes.

In accordance with the pioneering work of Vasunilashorn *et al*.,^28^ our investigation underscores the profound and enduring consequences of delirium, firmly aligning with their findings. Nonetheless, it is imperative to emphasize that our study encompasses a broader demographic spectrum, extending beyond those of advanced age who have undergone major surgical interventions, as was the case in their study comprising a cohort of 560 patients with a maximum follow-up duration of 3 years.^28^ Our study, in contrast, encompasses patients across various age groups, refuting the notion that postoperative agitated delirium and its potential association with future dementia is an exclusive concern of the elderly. Indeed, while advancing age undoubtedly serves as a significant risk factor for dementia,^30^ our extensive analysis reveals that the impact of postoperative agitated delirium on the development of future dementia is not confined to those aged 70 and above. In our comprehensive cohort, which comprises a notable proportion (approximately 30%) of patients aged below 65 (Supplemental Table 1), it is evident that postoperative agitated delirium remains a substantive risk factor for subsequent dementia, transcending the boundaries of age. This highlights the need for vigilance and proactive intervention in the broader population, as postoperative agitated delirium poses a sustained risk of future dementia, even in younger individuals. Building on this foundation, a meta-analysis conducted by Lee *et al*.^29^ that amalgamated findings from six distinct studies fortifies the notion that POD constitutes a tangible risk factor for the development of dementia. The cumulative patient population across these studies reached its zenith at 213, with a maximum follow-up period of 3 years, and it is worth noting that only one of these studies employed an ICD diagnosis for dementia.^29^ In contrast, our present study boasts a staggering total of 251 086 patients, meticulously matched through propensity score analysis to create two comparable cohorts, each consisting of 5466 patients. Our investigation, uniquely, extends the horizon with a comprehensive examination of the impact of postoperative agitated delirium on dementia, a facet hitherto unexplored in existing literature. Recognizing the potential influence of mortality on dementia incidence over an extended follow-up period, our study adopts a statistical approach to account for competing risks of death.^20,31^ Our rigorous analysis yields a compelling finding—a substantial and statistically significant association between postoperative agitated delirium and an increased risk of dementia. After meticulous adjustments, our study reports an aHR of 1.24 (95% CI, 1.07–1.45; *P* = 0.004) when comparing patients who experienced postoperative agitated delirium to those who did not, underscoring the clinical significance and relevance of our findings in understanding the long-term consequences of postoperative agitated delirium.

Our analysis revealed a substantial disparity in the cumulative incidence of dementia between patients who experienced POD agitation and those who did not, as depicted in Fig. 1B (*P* = 0.017). Among patients who underwent non-cardiac surgery before PSM, the 5-year cumulative dementia rates stood at 5.9% for the POD agitation group and 2.8% for the non-agitation group. After meticulous PSM adjustments to mitigate potential confounding factors, the 5-year cumulative dementia rates were 5.9 and 4.1%, respectively. Notably, our study pioneers the quantification of IR and IRR concerning the occurrence of dementia in patients with postoperative agitated delirium compared to those without postoperative agitated delirium. Specifically, the IR of dementia in patients with postoperative agitated delirium was calculated at 97.65 per 10 000 person-years, while the IR for patients without postoperative agitated delirium was 70.85 per 10 000 person-years. This yields an IRR of 1.21 (95% CI: 1.40–1.40), underscoring the heightened risk of dementia associated with postoperative agitated delirium compared to its absence. Importantly, this study represents a pioneering effort in establishing these IR and IRR metrics for postoperative agitated delirium across all age groups, a departure from previous research that primarily focused on older populations.

In recognizing the contributions of this study, it is crucial to address its inherent limitations. Firstly, it is essential to acknowledge the homogeneity of our study population, as only Asian participants were recruited. Thus, a degree of caution should be exercised when generalizing our findings to non-Asian populations, as there may be variations in patient demographics and healthcare practices. Secondly, our investigation hinges on a large-scale retrospective analysis utilizing a medical insurance database, comparing the occurrence of POD and its association with subsequent dementia development in patients undergoing non-cardiac surgeries. This retrospective nature, while harnessing the advantages of vast datasets, inherently carries the risk of information bias and residual confounding. Thirdly, remarkably, our study reports a lower occurrence of delirium compared to findings in previous studies. This difference might be because we specifically focused on patients with agitated delirium, excluding those with apathetic delirium from our analysis. Therefore, our findings pertain primarily to the risk of postoperative dementia in patients experiencing agitated delirium. Future research endeavours may necessitate a large-scale randomized controlled trial to comprehensively address the relationship between different subtypes of delirium and dementia risk. In spite of these limitations, it is important to underscore that our study benefits from a substantial volume of data and consistent baseline characteristics among participants. As such, the impact of these limitations on the overall robustness of our research results is likely to be minimal.

## Conclusion

In conclusion, our study provides compelling evidence that patients who experience postoperative agitated delirium are at a significantly elevated risk of developing dementia compared to those without such episodes. These findings underscore the critical clinical importance of both preventing and effectively managing postoperative agitated delirium in surgical patient care. The implications of our research extend beyond individual patient outcomes, carrying broader implications for healthcare policies and practices aimed at improving long-term cognitive health in surgical populations. Therefore, healthcare providers ought to incorporate strategies for preventing delirium and implementing early intervention protocols within perioperative care, providing a potential avenue for alleviating the substantial burden of dementia in the future. Further research into targeted interventions and their impact on dementia risk reduction is warranted, potentially offering new avenues for improving patient outcomes and enhancing the quality of healthcare delivery.

## Supplementary Material

fcae076_Supplementary_Data

## Data Availability

The study’s data, derived from the National Health Insurance Research Database (NHIRD), is restricted from public sharing due to the Personal Information Protection Act since 2012. Interested parties can seek access through a formal proposal, subject to governmental ethics review committee approval. For details on data access, visit http://nhird.nhri.org.tw/en/Data_Subsets.html#S3 and https://nhird.nhri.edu.tw/en/index.html. Dr. Szu-Yuan Wu, MD, PhD, ensures data integrity. Notably, the data has not been presented at scientific meetings. The study protocols were reviewed and approved by the Institutional Review Board of Tzu-Chi Medical Foundation (IRB109–015-B).
