# Supplementary material for: The impact of postoperative agitated delirium on dementia in surgical patients
Source: Brain Commun. 2024 Mar 19;6(2):fcae076. doi: 10.1093/braincomms/fcae076 (PMC10950050; doi:10.1093/braincomms/fcae076)
Supplement: fcae076_Supplementary_Data [file fcae076_supplementary_data.pdf]

Supplemental Table 1. Comparison of Characteristics between Postoperative Agitated Delirium and Non-Agitated Delirium Groups in Patients Receiving Non-Cardiac Major Surgery

	**Before propensity scores patching**					**After propensity scores patching**				
	**Non-Agitated Delirium**		**Agitated Delirium**		**ASMD**	**Non-Agitated Delirium**		**Agitated Delirium**		**ASMD**
	**N=245,619**		**N=5467**			**N=5466**		**N=5466**		
	**N**	**%**	**N**	**%**		**N**	**%**	**N**	**%**	
**Age** (mean±SD)	42.49 ± 18.96		44.52 ± 26.29		0.088	44.99 ± 23.77		44.52 ± 26.29		0.019
Age, median (IQR), years-old	41.00 (29.00,56.00)		48.00 (24.00,66.00)			49.00 (25.00,65.00)		48.00 (24.00,66.00)		
Age group, years					0.371					0.027
20-45	71,841	29.3%	1,647	30.1%		1,663	30.4%	1,646	30.1%	
46-55	69,420	28.3%	863	15.8%		812	14.9%	863	15.8%	
56-65	56,753	23.1%	1,211	22.2%		1,241	22.7%	1,211	22.2%	
>65	47,605	19.4%	1,746	31.9%		1,750	32.0%	1,746	31.9%	
**Sex**					0.279					0.017
Female	136,468	55.6%	2,283	41.8%		2,330	42.6%	2,283	41.8%	
Male	109,151	44.4%	3,184	58.2%		3,136	57.4%	3,183	58.2%	
**Income levels (NTD)**					0.375					0.048
Unemployment	1,948	0.8%	129	2.4%		114	2.1%	128	2.3%	
Financial dependent	69,746	28.4%	2,272	41.6%		2,385	43.6%	2,272	41.6%	
<2000	77,421	31.5%	1,656	30.3%		1,639	30.0%	1,656	30.3%	
2000-30000	48,663	19.8%	859	15.7%		811	14.8%	859	15.7%	
30000-45000	30,334	12.4%	371	6.8%		344	6.3%	371	6.8%	
＞45000	17,507	7.1%	180	3.3%		173	3.2%	180	3.3%	
**Urbanization**					0.097					0.016
Rural	63,514	25.9%	1,651	30.2%		1,692	31.0%	1,650	30.2%	
Urban	182,105	74.1%	3,816	69.8%		3,774	69.1%	3,816	69.8%	
**Surgical types**					0.866					0.041
Skin	3,947	1.6%	183	3.4%		198	3.6%	183	3.4%	
Breast	3,862	1.6%	29	0.5%		37	0.7%	29	0.5%	

Musculoskeletal	56,283	22.9%	906	16.6%		877	16.0%	906	16.6%	
Respiratory	11,010	4.5%	347	6.4%		346	6.3%	347	6.4%	
Digestive	50,744	20.7%	1,045	19.1%		1,015	18.6%	1,045	19.1%	
Kidney, ureter, bladder	77,579	31.6%	508	9.3%		494	9.0%	508	9.3%	
Neurosurgery	12,056	4.9%	1,053	19.3%		1,036	19.0%	1,052	19.3%	
Eye	7,012	2.9%	35	0.6%		33	0.6%	35	0.6%	
Others	23,126	9.4%	1,361	24.9%		1,430	26.2%	1,361	24.9%	
**Elective status**					0.340					0.052
Elective surgery	200,354	81.6%	3,658	66.9%		3,790	69.3%	3,658	66.9%	
Emergency surgery	45,265	18.4%	1,809	33.1%		1,676	30.7%	1,808	33.1%	
**ASA physical status**					0.586					0.037
1	158,507	64.5%	2,329	42.6%		2,333	42.7%	2,329	42.6%	
2	34,279	14.0%	822	15.0%		888	16.3%	822	15.0%	
3	42,734	17.4%	1,205	22.0%		1,177	21.5%	1,205	22.1%	
4	10,099	4.1%	1,111	20.3%		1,068	19.5%	1,110	20.3%	
**Types of anesthesia**					0.617					0.025
General anesthesia	152,472	62.1%	4,793	87.7%		4,837	88.5%	4,792	87.7%	
Regional anesthesia	93,147	37.920%	674	12.3%		629	11.51%	674	12.3%	
**Duration of anesthesia**					0.452					0.001
<= 3 hours	209,710	85.4%	4,180	76.5%		4,181	76.5%	4,180	76.5%	
>3 hours	35,909	14.6%	1,287	23.5%		1,285	23.5%	1,286	23.5%	
**Coexisting comorbidity**										
Diabetes	22,383	9.1%	845	15.5%	0.194	834	15.3%	845	15.5%	0.006
Hypertension	45,291	18.4%	1,727	31.6%	0.307	1,731	31.7%	1,727	31.6%	0.002
Hyperlipidemia	26,004	10.6%	797	14.6%	0.121	737	13.5%	797	14.6%	0.032
Coronary artery disease	19,920	8.1%	796	14.6%	0.205	746	13.7%	796	14.6%	0.026
Stroke	12,565	5.1%	1,058	19.4%	0.445	1,011	18.5%	1,057	19.3%	0.021
Depression	9,115	3.7%	474	8.7%	0.207	442	8.1%	473	8.7%	0.020
Anxiety	18,302	7.5%	613	11.2%	0.130	569	10.4%	613	11.2%	0.026
Heart failure	4,427	1.8%	300	5.5%	0.198	267	4.9%	300	5.5%	0.028

Peripheral vascular disease	5,410	2.2%	345	6.3%	0.205	330	6.0%	345	6.3%	0.011
Chronic Obstructive Pulmonary Disease	24,256	9.9%	890	16.3%	0.191	884	16.2%	890	16.3%	0.003
Atrial fibrillation	1,622	0.7%	132	2.4%	0.143	109	2.0%	132	2.4%	0.029
Traumatic head injury	0	0.0%	0	0.0%	0.000	0	0.0%	0	0.0%	0.000
Sleep disorder	17,190	7.0%	723	13.2%	0.330	721	13.2%	722	13.2%	0.001
Alcohol Liver diseases	4,176	1.7%	180	3.3%	0.102	172	3.1%	179	3.3%	0.001
Frailty	1,965	0.8%	65	1.2%	0.022	66	1.2%	65	1.2%	0.000
Vision or hearing impairment	2,751	1.1%	104	1.9%	0.017	101	1.8%	104	1.9%	0.001
Anemia	5,649	2.3%	235	4.3%	0.036	231	4.2%	235	4.3%	0.001
**Medications use**										
Diphenhydramine	27,755	11.3%	678	12.4%	0.007	675	12.3%	677	12.4%	0.001
Anticholinergics	21,124	8.6%	1,072	19.6%	0.339	1,069	19.6%	1,071	19.6%	0.001
Benzodiazepine	18,176	7.4%	1,011	18.5%	0.412	1,009	18.5%	1,010	18.5%	0.000
Gabapentinoids	16,211	6.6%	809	14.8%	0.320	805	14.7%	808	14.8%	0.001
Opioids	4,202	1.7%	115	2.1%	0.113	113	2.1%	115	2.1%	0.001
Other Psychotropic drug use	16,186	6.6%	704	12.9%	0.491	699	12.8%	704	12.9%	0.001
**CCI Scores**										
Mean (SD)	0.45 ± 1.14		1.01 ± 1.80		0.377	0.86 ± 1.54		1.01 ± 1.80		0.093
Median (IQR, Q1-Q3)	0.00 (0.00,0.00)		0.00 (0.00,2.00)			0.00 (0.00,1.00)		0.00 (0.00,2.00)		
CCI Scores					0.374					0.007
0	195,705	79.7%	3,447	63.1%		3,463	63.4%	3,446	63.0%	
≥1	49,914	20.3%	2,020	37.0%		2,003	36.6%	2,020	37.0%	
CCI										
Congestive Heart Failure	3,954	1.6%	273	5.0%	0.190	230	4.2%	273	5.0%	0.037
Chronic Pulmonary Disease	19,658	8.0%	762	13.9%	0.191	849	15.5%	762	13.9%	0.045
Rheumatic Disease	1,513	0.6%	42	0.8%	0.018	42	0.8%	42	0.8%	0.000
Liver Disease	20,886	8.5%	625	11.4%	0.098	728	13.3%	625	11.4%	0.057
DM with complications	4,489	1.8%	200	3.7%	0.112	187	3.4%	200	3.7%	0.013
Hemiplegia and Paraplegia	4	0.0%	0	0.0%	0.000	1	0.0%	0	0.0%	0.020
Renal Disease	4,052	1.7%	223	4.1%	0.146	233	4.3%	223	4.1%	0.009

Acquired Immunodeficiency Syndrome	102	0.0%	5	0.1%	0.020	1	0.0%	5	0.1%	0.030
Cancer	5,590	2.3%	449	8.2%	0.268	271	5.0%	449	8.2%	0.131
**Outcomes**					***P* Value**					***P* Value**
Dementia	12,251	5.0%	365	6.7%	<0.001	279	5.1%	365	6.7%	<0.001
Mean (SD) follow-up year	8.26 ± 4.96		6.84 ± 5.11		<0.001	7.42 ± 4.90		6.84 ± 5.11		<0.001
Median (IQR) follow-up year	8.22 (3.95,12.55)		6.18 (2.11,11.09)		<0.001	7.21 (3.07,11.47)		6.17 (2.11,11.08)		<0.001

Abbreviations: CCI (Charlson comorbidity index), SD (standard deviation), IQR (interquartile range), N (numbers), PSM (propensity score matching), ASA (American Society of Anesthesiologists), NTD (New Taiwan Dollars), ASMD (Absolute Standard Mean deviation).
